# Surface plasmon resonance imaging of excitable cells

**DOI:** 10.1088/1361-6463/aaf849

**Published:** 2019-01-04

**Authors:** Carmel L Howe, Kevin F Webb, Sidahmed A Abayzeed, David J Anderson, Chris Denning, Noah A Russell

**Affiliations:** 1 Department of Electrical and Electronic Engineering, University of Nottingham, Nottingham, NG7 2RD, United Kingdom; 2 School of Physics and Astronomy, University of Nottingham, Nottingham, NG7 2RD, United Kingdom; 3 Empyrean Therapeutics Ltd, Building 250, Babraham Research Campus, Cambridge, CB22 3AT, United Kingdom; 4 Department of Stem Cell Biology, Centre of Biomolecular Sciences, University of Nottingham, Nottingham, NG7 2RD, United Kingdom; da849em1 noah.russell@nottingham.ac.uk

**Keywords:** surface plasmon resonance, plasmon, cardiac, neuron, functional imaging, imaging, biosensor

## Abstract

Surface plasmons (SPs) are surface charge density oscillations occuring at a metal/dieletric interface and are highly sensitive to refractive index variations adjacent to the surface. This sensitivity has been exploited successfully for chemical and biological assays. In these systems, a surface plasmon resonance (SPR)-based sensor detects temporal variations in the refractive index at a point. SPR has also been used in imaging systems where the spatial variations of refractive index in the sample provide the contrast mechanism. SPR imaging systems using high numerical aperture (NA) objective lenses have been designed to image adherent live cells with high magnification and near-diffraction limited spatial resolution. Addressing research questions in cell physiology and pharmacology often requires the development of a multimodal microscope where complementary information can be obtained.

In this paper, we present the development of a multimodal microscope that combines SPR imaging with a number of additional imaging modalities including bright-field, epifluorescence, total internal reflection microscopy and SPR fluorescence microscopy. We used a high NA objective lens for SPR and TIR microscopy and the platform has been used to image live cell cultures demonstrating both fluorescent and label-free techniques. Both the SPR and TIR imaging systems feature a wide field of view (~300 *µ*m) that allows measurements from multiple cells whilst maintaining a resolution sufficient to image fine cellular processes. The capability of the platform to perform label-free functional imaging of living cells was demonstrated by imaging the spatial variations in contractions from stem cell-derived cardiomyocytes. This technique shows promise for non-invasive imaging of cultured cells over very long periods of time during development.

## 1. Introduction

Electrically excitable cells, such as cardiomyocytes and neurons, have been shown to produce fast optical signals that are the result of light scattering and birefringence changes associated with membrane depolarization [1–3]. In order to develop an understanding of how a population of cells are organized, exchange, and process information a new sensing technology is required, one that has single cell resolution over a relatively large network. Surface plasmon resonance (SPR) sensors possess highly sensitive resonance conditions, which make them capable of detecting variations in refractive index with a high spatiotemporal resolution, label-free thus enabling long-term recording.

**Figure 1.**
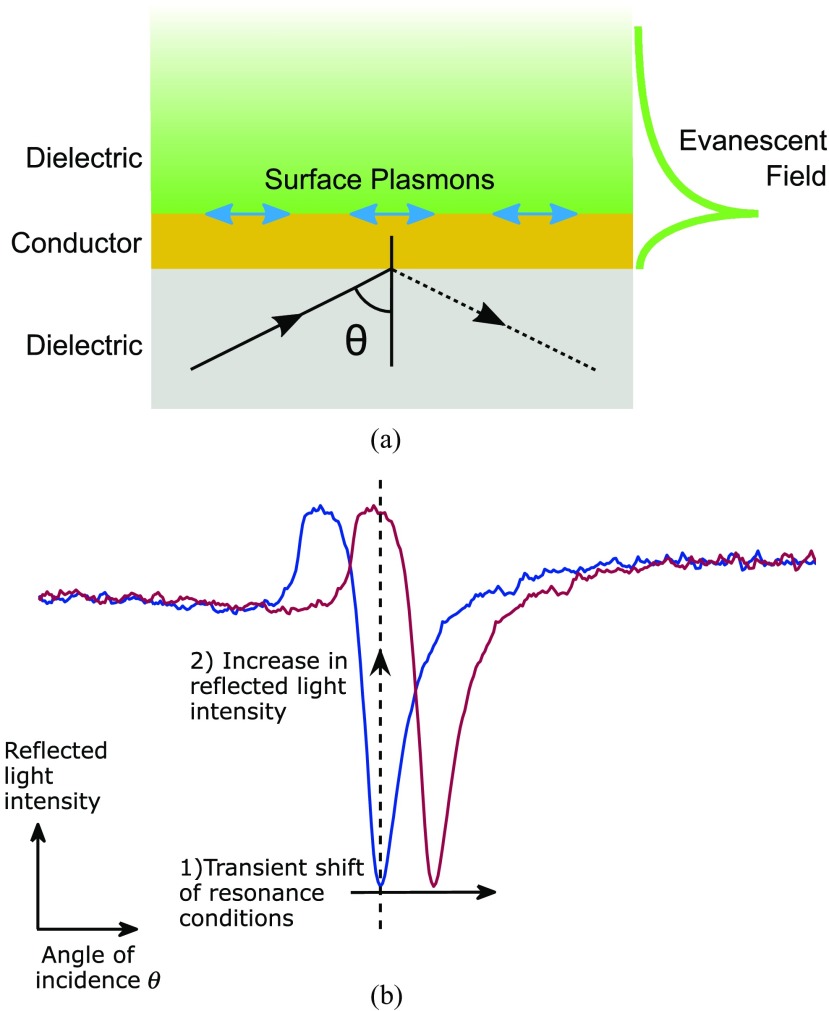
(a) SPR on a conductive layer. When resonance occurs SPs propagate along the interface between a conductor and dielectric. An evanescent field is generated that decays into both. (b) Diagram of angular and intensity modulation SPR sensor modalities. In SPR sensors with angular modulation, the change in resonance conditions, for example, the refractive index of dielectric, is detected by monitoring the change in the resonance angle, *θ*_sp_. In SPR sensors with intensity modulation, the angle of incidence is fixed and when *θ*_sp_ changes the detector sees an increase or decrease in light intensity.

SPR occurs when *p*-polarized light incident on a noble metal, at a specific angle of incidence, couples to the free electrons in the metal resulting in all the incident energy generating surface plasmons (SPs) [4]. At the angle of incidence where the plasmon coupling occurs, the intensity of the reflected light drops and an evanescent wave is generated in both the metal and dielectric (figure 1(a)). This evanescent wave penetrates into the dielectric to a depth of about 150 nm, depending on the properties of the metal used [5, 6]. SPR is very sensitive to perturbations of refractive index within this evanescent field; therefore, when there is a change in the refractive index of the dielectric medium, the characteristics of the light wave coupled to the surface plasmon changes and the resonance conditions are altered (figure 1(b)). This allows SPR to be used for label-free imaging of both structural (sensitive to local variations in density) [7–10], and functional (sensitive to movements of matter) [11–14] features.

SPs cannot be excited by the incident light directly. At a given photon energy (*hω*) the wave-vector of the incident *p*-polarized light must be increased so the photons can be coupled into plasmons [15]. Passing the illumination light through a high refractive index dielectric material, such as glass, can shift the wave-vector of the incident light enough to excite SPs. Excitation methods based on attenuated total reflection (ATR) were demonstrated in the late sixties by Otto [16] and Kretschmann and Raether [17]. The Kretschmann ATR configuration is one of the most popular methods for exciting SPs and has a thin metal film deposited on top of a prism surface.

The Kretschmann–Raether configuration allows a large field of view (FOV) (up to cm). However, this configuration is not well suited to imaging because it requires a very shallow angle of incident light. The physical constraint of the prism limits the numerical aperture (NA) and magnification of the imaging system resulting in the configuration having a reduced spatial resolution [18]. Additionally, the FOV is angled relative to the objective resulting in an anisotropic distortion. Using a high NA objective lens instead of a prism to shift the wave-vector of the incident light resolves the issues of spatial distortion by keeping the object plane parallel to the image plane [18, 19]. Using a high NA objective also allows higher magnification and, because of the high NA, the spatial resolution is much improved and can be diffraction limited (~300 nm).

Despite these caveats, using SPR for functional imaging by detecting refractive index variations within the evanescent field is possible. To detect variations in the refractive index of the dielectric medium interfaced to the metal surface, a number of detection schemes can be employed: (i) by tracking the angle of minimum light intensity (angular modulation) [20]; (ii) by monitoring the intensity with the angle of incidence fixed at the position with the steepest gradient on the SPR curve (intensity modulation) [21, 22] or using differential intensity detection approaches [23, 24]; (iii) by detecting the phase of the reflected light [25]. The detection of small refractive index changes over a relatively large volume has been successful on some sensors based on an intensity modulation scheme down to a sensitivity (Δ*n*_*min*_) of 10^−6^ refractive index units (RIUs) [21, 22]. Better sensitivity levels have been achieved using other detection methods down to 10^−7^ RIUs using angular modulation [20], however, it is experimentally more complicated to monitor the angle of minimum reflection at fast sampling rates. A trade-off between sensitivity and sampling rate will be necessary if SPR is to be used for fast, functional imaging.

The spatial resolution of the SPR imaging system is limited in the direction perpendicular to the propagation of the SP [26]. Therefore, complementary information can be obtained from other methods including total internal reflection microscopy (TIRM). TIRM sees an evanescent wave generated at an interface separating two media with different refractive indices when the incident light is totally internally reflected (see supplementary information (stacks.iop.org/JPhysD/52/104001/mmedia)) for discussion on total internal reflection). Discontinuities in the refractive index of the cell membrane give rise to differences in the reflected light intensity. TIRM has been used to study cell-surface interactions for improved drug delivery systems [27].

The evanescent wave generated at the interface is capable of exciting fluorophores, and since the intensity of the evanescent field decays exponentially with distance from the interface, only fluorophores within ~100 nm are excited. In comparison with standard epi-fluorescence the background is 2000 times lower because no out of focus fluorescence gets excited thus resulting in a high signal-to-noise ratio (SNR) [28]. Depending on the substrate this technique is known as total internal reflection fluorescence microscopy (TIRF) [29, 30] or SPR fluorescence microscopy (SPRF). TIRFM has not been demonstrated in this study, however, we have shown the capability of the imaging system to excite fluorescence using SPR. SPRF takes advantage of the enhanced electromagnetic field intensity that occurs when plasmons are excited, resulting in amplified fluorescence signals (see [31] for a detailed review).

Applying SPR imaging to research questions in cell physiology and pharmacology requires the development of a multi-modal system where complementary information can be obtained. Information acquired from label-free techniques, such as SPR and TIRM can be validated with well established fluorescent labelling methods. Likewise, the system can be used to validate the functional imaging capabilities of SPR.

In this paper, we present a multimodal imaging platform that includes SPR microscopy using a high NA objective applied to live cell imaging with the following capabilities: (i) the SPR system features a wide FOV providing the ability to study ~40 cells simultaneously, with subcellular resolution. (ii) The SPR system is used to image neuronal cells while resolving axons and dendrites. We comment on the factors that affect the resolution of fine neuronal processes. (iii) We show that complementary information on the imaging resolution can be obtained from TIRM and demonstrate a combination of microscopy systems including bright-field, epi-fluorescence and SPRF that can be applied to cell physiology. (iv) We combine a number of the imaging techniques to demonstrate a typical experiment where structural and functional imaging is desired and in doing so we verify the ability of the system to study spatiotemporal cellular functions by imaging localized contractions of stem cell-derived cardiomyocytes. (v) We describe a detailed design of this platform to enable ease of implementation and characterization for use by the cell physiology community.

## 2. Methods

### 2.1. Optical system design

**Figure 2.**
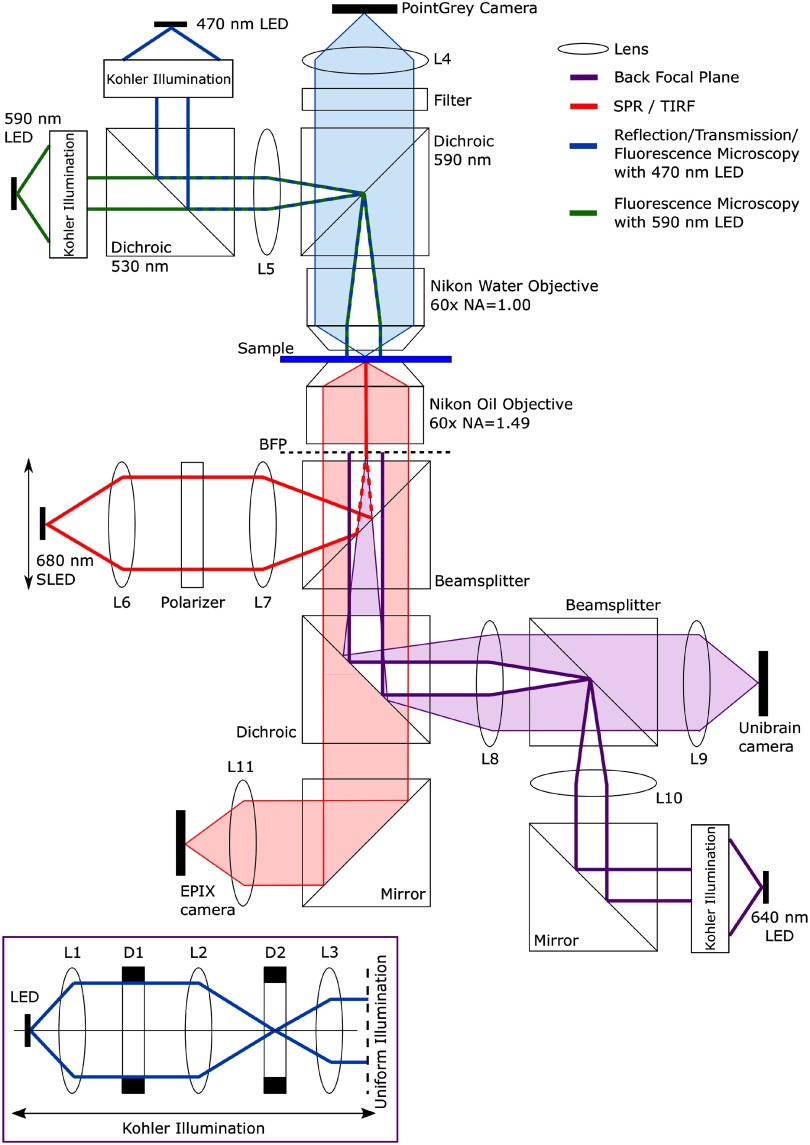
Schematic of the SPM that was developed. Several imaging modalities can be simultaneously exploited—including bright-field, epi-fluorescence, TIRFM, and SPR imaging. Each optical pathway has been diagrammed within the figure. Imaging pathways are shaded, while the illumination pathways are shown with bold lines. Electrophysiology micro-manipulators (not shown) are also incorporated to provide for validation and calibration of the SPR signal with intracellular electrical activity.

The custom-built multimodal imaging system we have developed combines SPR imaging with a number of microscopy sub-systems that include bright-field, epi-fluorescence, TIRM and SPRF. Figure 2 shows a schematic of the optical system and optical pathways. The system is also capable of performing electrophysiological measurements simultaneously using microelectrode technology. The rig was mounted on a floating optical table (Thorlabs, Newton, NJ, USA) to minimize mechanical perturbations, to which such sensitive methods are highly susceptible.

**Figure 3.**
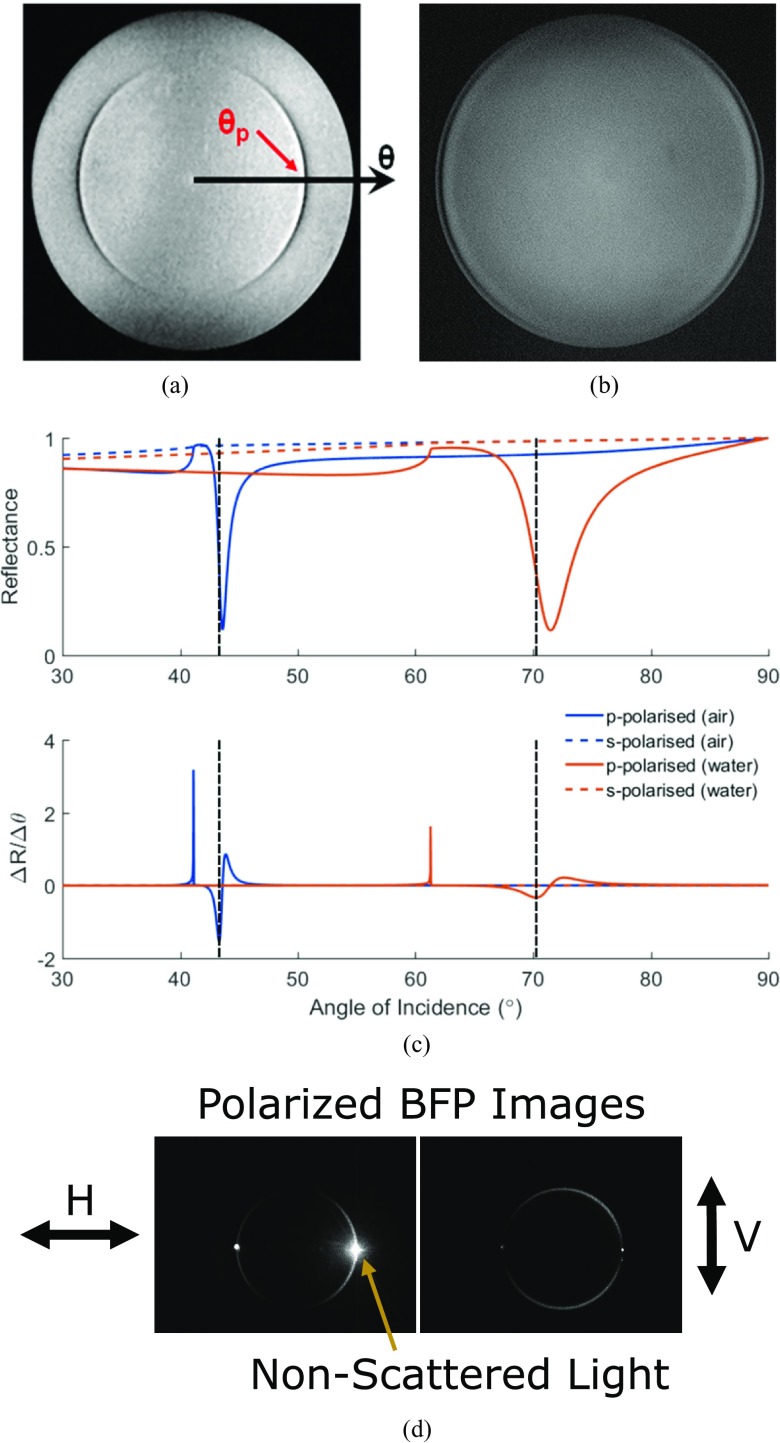
Images of a uniformly illuminated BFP, with the gold sample in air (a) and water (b). The dark arcs indicate the angles at which plasmon resonance occur. Note that only *p*-polarized light can excite plasmons, which is why arcs appear and not a ring. (c) Theoretical angular SPR response for *p*- and *s*- polarized light with air and water as the dielectric. *S*-polarized light is not capable of exciting SPs. Using *p*-polarized light and increasing the refractive index of the dielectric increases the angle where resonance occurs. The angle where the gradient (Δ*R*/Δ*θ*) is greatest is marked on each curve. (d) Images of the BFP imaged through a polarizer with cells growing on a gold SPR surface. Note that light scattered off refractive index discontinuities in the sample returns from the same elevation angle but all azimuthal angles. This illustrates that momentum is conserved during plasmon scattering.

A 640 nm light emitting diode (LED) was used in conjunction with Köhler illumination to illuminate the back focal plane (BFP) of the Nikon Oil Objective lens (CFI Apo TIRF 60×, NA  =  1.49, oil-immersion lens, Nikon Inc., Tokyo, Japan) with a uniform light intensity [32]. The BFP was monitored on a complementaty metal oxide semiconductor (CMOS) camera (Unibrain, 640  ×  480 pixels, 5.6 *µ*m pixel size, Fire-i^™^, Unibrain Inc., San Ramon, CA, USA), shown in figure 3(a).

Reflection microscopy was performed with the 470 nm LED uniformly illuminating the BFP of a water-dipping objective (60×, NA  =  1.00, Nikon Inc, Tokyo, Japan). The reflected light passed through the same objective lens, and was imaged on to a CCD camera (Grasshopper3, 1920  ×  1440 pixels, 4.54 *µ*m pixel size, S3-U3-28S5M-C Point Grey, Richmond, BC, Canada). Transmission microscopy was performed using the 470 nm LED and the transmitted light was imaged onto a second CMOS camera (640  ×  480 pixels, 9.9 *µ*m pixel size, SV643M, EPIX Inc., Buffalo Grove, IL, USA). The 470 nm and 590 nm LEDs could also be used for exciting fluorescence, in combination with appropriate emission filters.

TIRM was achieved using the 640 nm LED, in conjunction with Köhler illumination to uniformly illuminate the BFP of the objective. The aperture diaphragm (D1) within the Köhler illumination was closed so that contrast was obtained in reflection by frustration of the critical angle (see supplementary information for information on the critical angle). The reflected light was imaged on the Unibrain camera.

When the objective pupil is filled with plane-polarized light from the 640 nm LED (figures 3(a) and (b)), in the sector where the azimuthal angle is 0° the polarization state is pure *p*-polarized light, while pure *s*-polarized light arrives at the sample where the azimuthal angle is  ±90°. The dark arcs visible in figure 3(a) show the angle of incidence where the light is coupled into SPs and is therefore no longer being reflected. There is no dark band in the vertical direction (±90°) due to the light being pure *s*-polarized, and therefore incapable of exciting SPs.

SPRF was performed by diffusional loading of Alexa Fluor^®^, 680-dextran (3kDa, Invitrogen^™^, D34681) into the cytoplasm of the cell of interest, using a micropipette attached to the cell soma. The fluorescent dye was then excited using the 680 nm superluminescent LED (SLED, 1 mW, Superlum Diodes Ltd, Cork, Ireland) which was tuned to excite plasmons beneath the cell of interest. The SPR fluorescence was imaged on the Point-grey Grasshopper3 camera. SPRF microscopy could be performed to confirm that the cells on the sample were in the evanescent field prior to functional SPR imaging.

The SPR imaging system was developed around a high NA (NA  =  1.49) objective lens and is able to exploit both angular and intensity modulation by either shifting the illumination angle (laterally translating the focal spot in the BFP) or holding this angle constant at the angle of maximum SPR gradient and monitoring the resulting intensity.

Angular modulation uses monochromatic light to excite the SPs, and the excitation can be seen as a dip in the angular spectrum. The detector senses a shift in the angle where the reflected light intensity is at a minimum. Angular modulation was achieved using the 680 nm SLED, generating light that is focused onto the objective BFP, resulting in an illuminating beam at the sample of an adjustable, narrow range of angles. A SLED was used over a laser or LED because they have a high spatial coherence but a low temporal coherence, allowing the light to be focused to a very tight spot [33, 34], without suffering from the effects of laser speckling [35]. Additionally, SLEDs have extremely low noise [36], which is advantageous in the high-speed acquisition of rapid signals such as electrical activity in cells.

The output of the fiber-coupled 680 nm SLED was collimated using an achromatic lens (*f*  =  100 mm) and passed through a polarizer so that only *p*-polarized light was incident on the sample. The polarized light was focused to a diffraction-limited spot on the BFP of the objective lens with a second achromat lens (*f*  =  75 mm). The diffraction-limited spot, along with the polarizer, reduces the level of background caused by the *s*-polarized light that is unable to excite SPs. The radial position of the illumination spot in the BFP dictates the angle of incidence at the sample. The light scattered off refractive index discontinuities in the sample when cells are cultured on the gold coverslips returns from the same elevation angle but all azimuthal angles. This illustrates that momentum is conserved during plasmon scattering, as shown in figure 3(d). To change the angle of incidence at the sample the focused SLED beam was scanned across the BFP of the sample using a stepper motor traveling at 0.1 mm s^−1^. The reflected light was imaged on the EPIX camera at 24 fps, with an exposure time of 0.124 ms to provide reflection values as a function of the angle of incidence. Regions of interest were selected on the surfaces using Image-J and the *z*-axis profile was exported to MATLAB (figure 3(c)).

SPR sensing with intensity modulation (IM) works by fixing both the angle of incidence and wavelength and measuring the strength of the coupling between the light wave and the SP; detection is achieved by measuring the change in the intensity of the reflected light (figure 1(b)). Therefore, for IM detection the angle of incidence (*θ*) was fixed at the angle with the steepest gradient, Δ*R*/Δ*θ* (around 30% of the minimum intensity recorded at the trough of the SPR dip, marked on figure 3(c)) and the variation of the reflected light intensity was monitored on the EPIX camera.

### 2.2. SPR sensor preparation

The glass coverslips (19 mm, Karl Hecht GmbH and Co KG, Sondheim, Germany) were treated with (3-mercaptopropyl) tri-methoxysilane (MPTS, Sigma Aldrich, 174617) to present thiol groups prior to thermal metal evaporation [37]. MPTS was used instead of the usual chromium or titanium adhesion layer to preserve the surface plasmon quality [38]. Silanisation was performed immediately after solvent and plasma cleaning. The plasma cleaned coverslips were immersed in a boiling solution of 50:1:1 isopropanol: MPTS: deionised water for 10 min, then rinsed with isopropanol, dried under nitrogen and transferred to an oven at 100 °C for 10 min. The boiling, rinsing and baking steps were repeated three times.

The MPTS glass coverslips were coated with ~50 nm thick gold by thermal metal evaporation. Gold evaporation was carried out using a thermal evaporator (E306A, Edwards, Burgess Hill, UK). Gold wire was placed in a filament and the chamber was pumped down to 10^−1^ mbar with a rotary pump, then to 10^−7^ mbar with an oil diffusion pump, while being cooled with liquid nitrogen. A current of ~30 A was passed through the filament, so the metal melted and evaporated onto the glass coverslips held above the source with rare earth magnets. An Intellemetrics IL100 quartz crystal microbalance was used to estimate the evaporation rate and film thickness. The thickness of the gold coverslips was measured with a spectroscopic ellipsometer (Alpha-SE, J.A. Woollam, Lincoln, NE, USA) at a fixed incident angle (70°) and with a wavelength range between 380 to 900 nm.

### 2.3. Sensitivity experiments

The sensitivity of the planar gold surfaces was experimentally determined using sodium chloride (NaCl) salt-water solutions. Different concentrations of salt solutions were manually added to a chamber on top of a gold-coated coverslip using a 1 ml pipette whilst the reflected SPR light intensity response was measured. Deionized water was added as the first sample to find the reflection gradient maximum and the SLED was positioned here for the entirety of the experiment. While keeping all other parameters fixed, the refractive index of the media was adjusted using different concentrations of salt solution. The change in refractive index was calibrated with an Abbe Refractometer (Abbemat 200, Anton Paar, Graz, Austria). The reflected light from the sample plane was recorded using the EPIX camera (12-bit) at ~286 Hz. To allow the solution to stabilize, 30–60 s was left between each step. The resulting intensities were exported using Image-J and further analysis was performed in MATLAB.

### 2.4. Substrate preparation

For cell-based experiments on glass, the coverslips were treated with poly-L-lysine (PLL, Sigma Aldrich, P4707). The PLL coated glass coverslips were prepared by immersion in PLL solution for 5 min followed by thorough rinsing with deionized water.

For cell-based experiments on planar gold, the coverslips were treated with 11-Amino-1-undecanethiol (AUT, Sigma Aldrich, 674397), to generate a self-assembled monolayer [39]. First, the gold coverslips were solvent cleaned, followed by an oxygen plasma treatment.

The AUT was dissolved in ACS grade Ethanol at 1 mM, which was then poured into an immersion cylinder to completely cover the gold. Immersion time was not less than 18 h. Following immersion, the solution was drained from the cylinder and the gold was thoroughly rinsed, first with ethanol to remove the bulk AUT solution, and then with distilled water to remove the cytotoxic ethanol. The coverslips were dried thoroughly with N_2_ before cell plating.

### 2.5. Cell culture

The 3T3 fibroblast cell line was maintained in Dulbecco’s Modified Eagle Medium (DMEM, Gibco, 11960) with 10% fetal bovine serum (FBS, Gibco, 10270). Cells were subcultured and seeded on to AUT treated gold coverslips at a density of 0.3  ×  10^6^ cells.

The stem cell-derived cardiomyocytes were differentiated on AUT treated gold coverslips from human embryonic stem cells (hESC-CM) following the protocol in [40].

Cultures of primary hippocampal neurons were dissected from E18 Wistar rat embryos following a standard protocol [41]. The primary rat hippocampal neurons were plated on the functionalized coverslips with ~150 000 dissociated cells in 500 *µ*l media.

## 3. Structural imaging

### 3.1. Optical system characterisation

#### 3.1.1. Field of view (FOV).

Functional imaging of excitable cells using SPR microscopy requires performing measurements from a number of single cells to inspect the cell–cell interactions and to replicate single cell measurements. Therefore, the system has been designed with a wide-FOV to allow imaging of multiple single cells with subcellular resolution. This has been achieved by deliberately reducing the magnification of the imaging system.

The FOV was increased by mismatching the objective and tube lens to reduce the effective magnification of the 60×  oil-objective. The Nikon objective lens is designed for a tube lens with a focal length of 200 nm, however, we used a 60 mm tube lens. This reduces the effective magnification to 18×  and enlarges the FOV to up to 500 *µ*m, depending on the size of the camera sensor.

**Figure 4.**
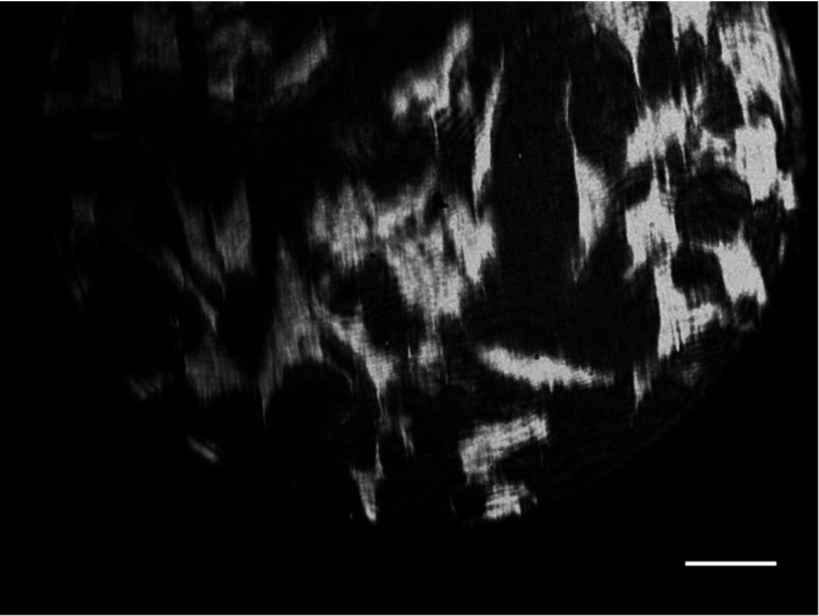
SPR image of cultured fibroblasts indicating high contrast over a wide FOV (350  ×  250 *µ*m). The scale bar is 40 *µ*m.

The number of pixels in the EPIX camera sensor is 640  ×  480, at 9.9  ×  9.9 *µ*m each, so the FOV has been increased to 350  ×  250 *µ*m from 105  ×  80 *µ*m. However, the back aperture of the objective lens restricts the usable FOV to a circular diameter of 320 *µ*m. Figure 4 shows SPR images of mouse fibroblast cells (3T3) seeded on gold coverslips, showing the wide FOV and the potential to image relatively large populations of cells, for example, there are ~40 cells within figure 4.

#### 3.1.2. Spatial resolution.

The resolution of a conventional light microscope is limited by the diffraction limit [42]. The theoretical diffraction-limited resolution of the optical imaging modalities presented (transmission and reflection microscopy) for a 60×, 1.49 NA microscope objective with 680 nm incident light given by the Abbe limit is 0.28 *µ*m. However, by under magnifying to 18×, the minimum resolvable feature, magnified onto a 2  ×  2 region of the sensor in accordance with the Nyquist criteria, is 1.1 *µ*m. The average measured size of a typical cultured mammalian neuronal soma is ~16 *µ*m (data not shown) [43] and a typical cardiomyocyte cell is about 100 *µ*m long and 10–25 *µ*m in diameter [44]. Even after undersampling the resolution is still more than adequate for imaging both cardiomyocytes and networks of neurons including many of the larger dendritic and axonal processes which are typically 1–2 *µ*m in size [45].

**Figure 5.**
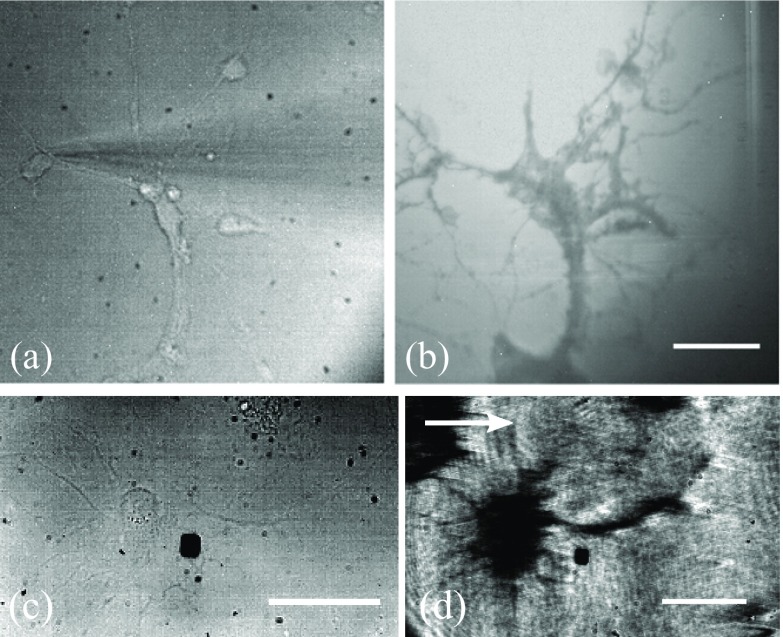
Transmission (a) and (c), TIRM (b) and SPR (d). (a), (b) Images of primary hippocampal neurons cultured on glass surfaces. (a) The glass surface was illuminated with the 470 nm LED and imaged on the EPIX camera. A microelectrode is visible in the image. (b) The 640 nm LED uniformly illuminated the BFP of the objective. The light was stopped down to close to the TIR critical angle to gain TIRM contrast. The scale bar in (b) is 40 *µ*m long and consistent across (a) and (b). (c), (d) Images of primary hippocampal neurons cultured on gold surfaces. (c) The gold surface was illuminated with the 470 nm LED and imaged on the EPIX camera. (d) SPR image of a neuron cultured on the SPR sensor. Arrow indicates the direction of plasmon propagation. The scale bars in (c) and (d) are 40 *µ*m long.

The resolution in SPR sensors, however, is determined by the propagation length of the SPs. The plasmons propagate along the metal/dielectric interface for a distance decided by the propagation length before they decay back into photons. This limits the resolution of the system in the direction parallel to the propagation of SP waves. So, when the propagating plasmon reaches a refractive index boundary it is no longer supported and is re-radiated as a photon. This means that even though in theory the plasmon propagation length in water on a 50 nm gold film is typically ~3.1 *µ*m [18], fine details, such as neuronal axons and dendrites can be resolved. Figure 5(c) shows a primary, hippocampal neuron cultured on a gold sensor imaged at 5 d *in vitro* (DIV). The fine, elaborating features visible in the figure demonstrate that axons and dendrites can be clearly resolved using SPR imaging in this regime. However, cell processes that are perpendicular to the propagation of the SPR are hardly resolved, due to the resolution limit of the SPR system, while they are clearly visible when they are parallel to the propagation of the SP wave [26].

To demonstrate the resolution limitations of the SPR microscopy, we explored an alternative evanescent wave microscopy technique that is based on total internal reflection (figure 5(b)). Primary hippocampal neurons were cultured on glass coverslips treated with PLL. The glass surface was illuminated with a uniform disc of light from the 640 nm LED source objective BFP. This disc of light was stopped down using the aperture diaphragm (D1) to just above the total internal reflection critical angle to gain contrast.

Figure 5(b) shows that TIRM can resolve dendrites in both directions demonstrating a superior resolution compared to SPR. Although TIRM provides resolution and contrast based on the frustrated total internal reflection, it has a limited ability to resolve functional time-resolved information compared to SPR.

#### 3.1.3. Refractive index mapping.

In biological samples, spatial differences in the lipids and proteins present result in a variation of the refractive index. These differences provide the contrast in an SPR image because as previously explained, refractive index discontinuities in the sample result in the propagating plasmons being re-radiated as photons.

**Figure 6.**
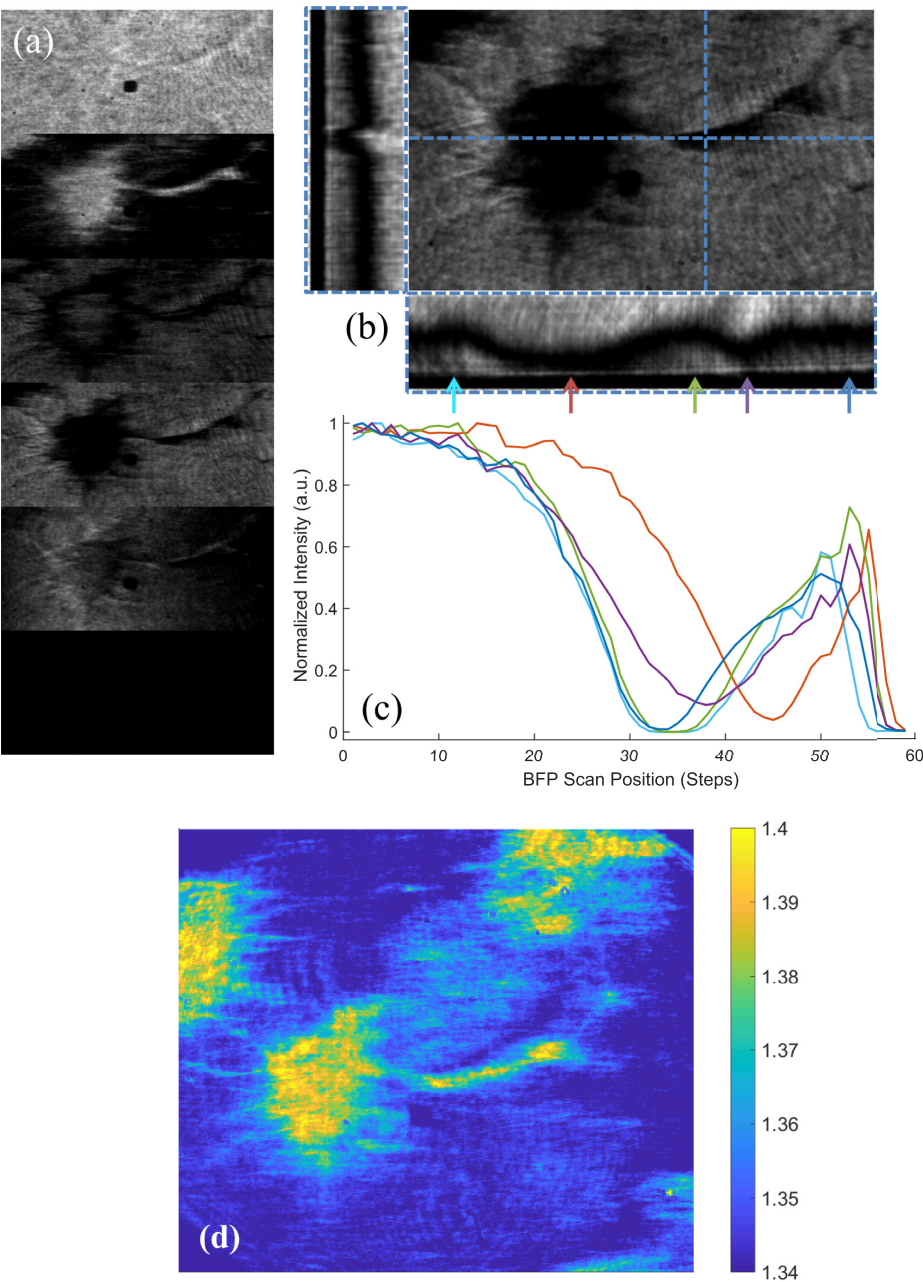
(a) Successive adjustments of SPR excitation by varying the angle of incidence of the SLED—top to bottom: excitation below the SPR angle (*θ*  <  63°), SPR excitation on the coverslip (*θ*  ≈  71°), middle of SPR dip on the cell (*θ*  ≈  75°), SPR excitation occurring under the cell (*θ*  ≈  79°), edge of the BFP (*θ*  ≈  80°), outside the BFP (*θ*  >  80°) (dark). (b) BFP angle-scan, with vertical and horizontal projections of the stack—dark line represents the SPR dip, with the ‘*Z*’ axis encoding the BFP angle. The scale bar in (a) and (b) is 10 *µ*m long. (c) Angle scan at various points across the cell and coverslip, extracted from the projection in (b), indicated by arrows. (d) Pseudo-color image showing the effective refractive index (in RIU) at each pixel in the FOV. This was created by scanning the illumination angle and finding the angle of minimum reflection for each pixel. The effective refractive index is reasonably homogeneous underneath the soma indicating the cell has adhered to the surface uniformly.

To assess the spatial variation of the refractive index on our gold surfaces, the refractive index of each pixel in the FOV of figure 5(d) was determined by scanning the illumination angle, figure 6(a) and finding the angle of the minimum reflection for each pixel, figures 6(b) and (c). Then by solving the Fresnel equations for reflection and transmission in a multi-layer device, the refractive index was calculated (see supplementary information).

Figure 6(a) shows the full image at various angles during the scan. The reflection as a function of scan angle at one *x*-position and one *y*-position was determined and shown in figure 6(b). The dark line in the projections represents the SPR dip and corresponds to the angle of incidence at the gold layer. The complete angle scan is shown at various points on the sample in figure 6(c). Note that at different angles of incidence plasmons are excited successively either under the cells or on cell-free background areas.

Figure 6(d) shows that the effective refractive index is reasonably homogeneous underneath the soma indicating the cell has adhered to the surface uniformly. The effective refractive index in a multilayer system is a function of the gap distance between the SPR sensor and the cell membrane and is weighted with an exponential decay [8, 10]. A larger effective refractive index indicates that the cell membrane has closely adhered to the surface, conversely a smaller measured refractive index indicates that the cell membrane is further away from the surface. Cell membranes are composed mostly of lipids and proteins which have refractive indices in the range of 1.46–1.54, so the refractive index we have measured here of 1.40 for the cell structure is less than previously reported values [46, 47]. The lower RI can be attributed to the lower RI cytosol (*n*  =  1.35 [46]) reducing the effective RI alongside the gap distance. It is important that the gap distance is less than the evanescent field distance and this will be addressed next.

### 3.2. SPR-excited fluorescence imaging

**Figure 7.**
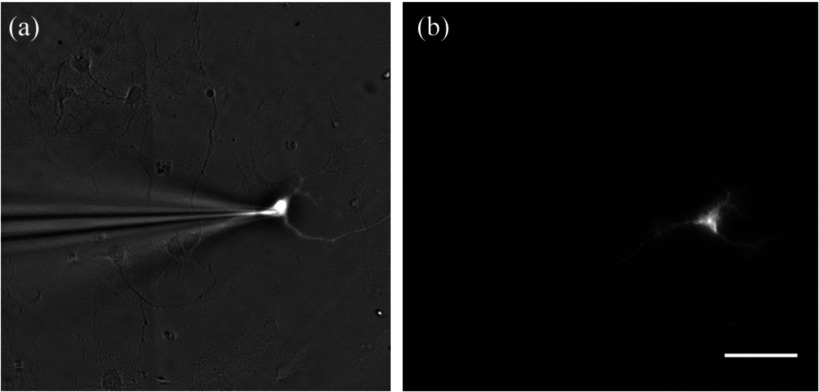
Images of a cultured neuron on an SPR sensor. (a) A microelectrode filled with a fluorescent dye is patched to a neuron and imaged using reflection microscopy, so the excited dye can be seen under epi-fluorescent illumination. (b) A neuron filled with fluorescent dye illuminated with SPR. This confirms the neurons are closely adhered to the gold SPR surface and within the evanescent field.

As described in the introduction, the evanescent field generated at the interface can excite fluorophores placed within it. Exploiting this phenomenon, we were able to determine whether the neurons were close enough to the gold surface for their membranes to lie within the evanescent field by patching a microelectrode filled with dye into the cell. The dye within the cell was excited using both epi- and SPR-excited fluorescence, figures 7(a) and (b), respectively. Figure 7(b) confirms that the 680 nm SLED SPR system can excite the dye-labeled cell and therefore, the cell is within the evanescent field. This is a necessary prerequisite to allow functional imaging using SPR. 100% of cells tested (*N*  =  12) were found to lie within the evanescent field. This demonstrates that SPR can be used to excite either structural or functional fluorescent dyes in studies of neuronal (or other) cultures.

## 4. Functional Imaging

For label-free functional imaging of dynamic, cellular functions from living cells, a low noise, high sensitvity system is required. We characterized this sensitivity by changing the bulk refractive index of the dielectric on a gold surface. This characterization is followed by results that demonstrate the ability of the system to image localized dynamic processes by detecting spatial variations in the contraction of stem cell-derived cardiomyocytes.

### 4.1. Sensitivity to refractive index changes

The sensitivity of the gold surfaces on the SPM was experimentally determined by altering the bulk refractive index of the dielectric on a 50 nm planar gold sample.

The concentration of the solution on the surface on the substrate was changed to either increase or decrease the refractive index using NaCl solutions. The concentration values were chosen so that the refractive index increase or decrease was in step sizes of Δ*n*  =  0.1 × 10^–3^. To find the sensitivity, the angle of incidence was chosen such that the reflection gradient (Δ*R*) is at its maximum. Practically, it is difficult to set this parameter, so the dynamic range was first established by angle-scanning across the SPR dip, and setting the SPR illumination angle to the location of the greatest gradient (figure 3(b)). There is a trade-off between greater sensitivity and SNR, as at the angle of the maximum gradient the light intensity is much lower, reducing the Poisson-limited SNR. Considering this, the angle of incidence was fixed at around 30% of the dynamic range.

**Figure 8.**
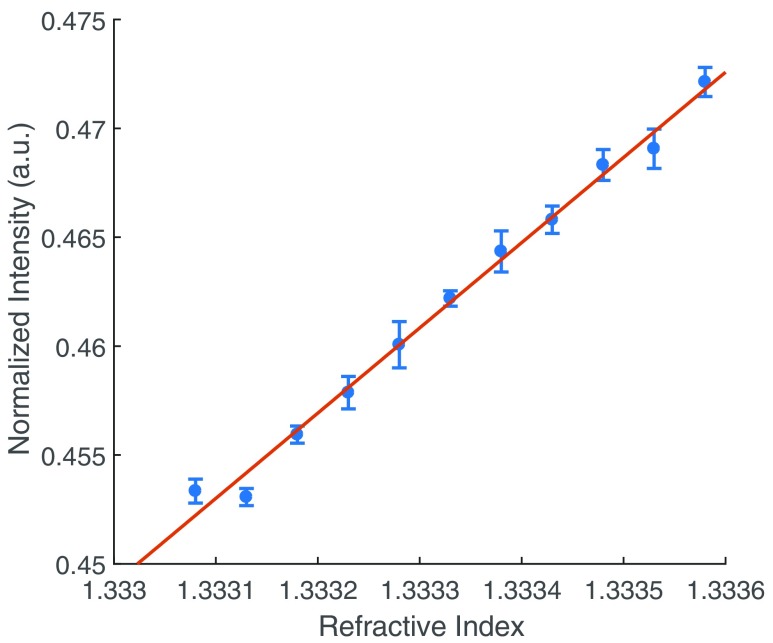
The sensitivity of the SPR imaging system. The refractive index was adjusted using a series of different concentration NaCl solutions. A 1 mW 680 nm SLED light source was used with a 12-bit CMOS camera and a 3.5 ms exposure time. The sensitivity in RIU was calculated as 2  ×  10^−5^. The linear fit is $y=({{p}_{1}}\times {\rm RI})+{{p}_{2}}$, where ${{p}_{1}}$  =  39.137 and ${{p}_{2}}$  =  −51.72. *R*^2^  =  0.9913.

The results from all the experiments are summarized in figure 8. Figure 8 shows that the normalized light intensity (Δ*R*) increases linearly with increasing refractive index. The sensitivity of the sensor was characterized by the RIU as 2  ×  10^−5^.

The experimentally measured sensitivity here is less than the value of ~10^−6^ RIU reported previously from intensity modulating SPR techniques in literature [21, 22]. However, the level of detection in these sensitivity experiments is limited by the exposure time because the power of the noise is proportional to the bandwidth. So, for the relatively small bandwidth used for these measurements (286 Hz), the total noise power may be reduced if a lower sample frequency is used thus increasing the SNR.

### 4.2. Detection of cardiomyocyte contraction

**Figure 9.**
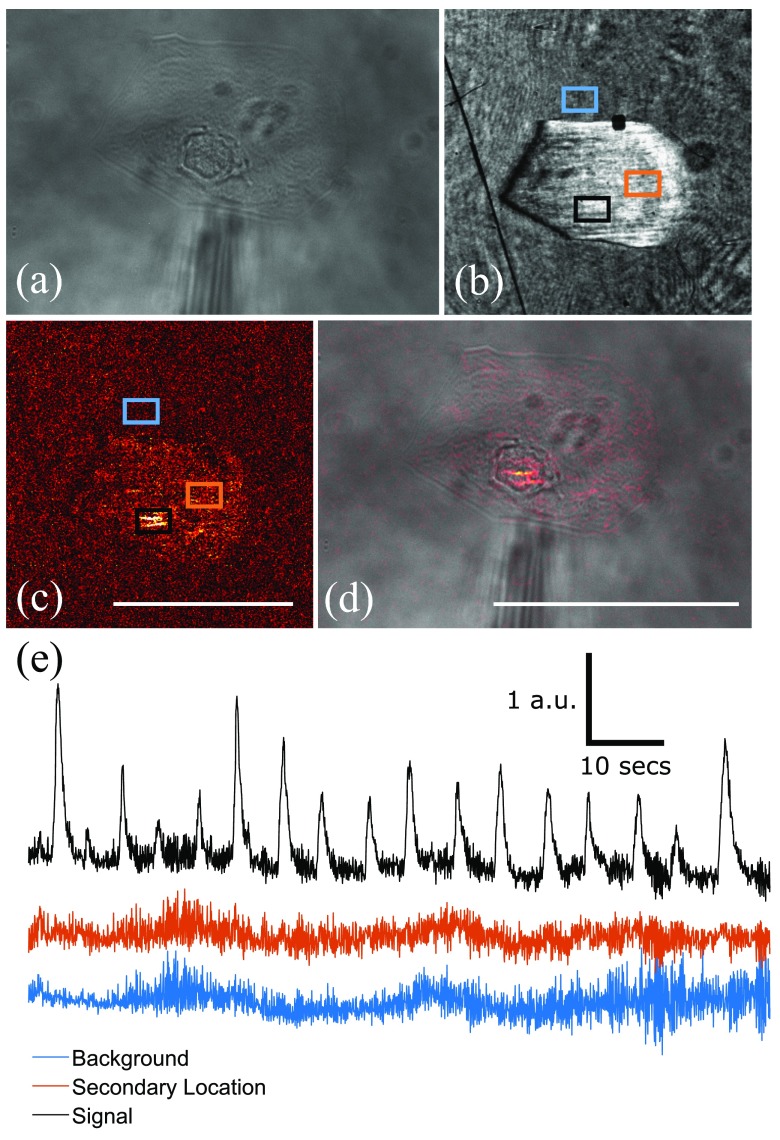
(a) Bright-field image of a stem cell-derived cardiomyocyte taken using reflection microscopy. (b) SPR image of the cell from (a). (c) There are two stripes of localized light intensity changes, which can be seen when subtracting the first frame of the SPR response from the remaining image stack. The scale bar is 10 *µ*m long and consistent across (b) and (c). (d) Overlay of (c) on (a). The area of the greatest light intensity change appears to be around the nucleus of the cell. The scale bar is 10 *µ*m long and consistent across (a) and (d). (e) Relative change in the light intensity of the SPR signal from a beating stem cell-derived cardiomyocyte, a control area directly next to the cell of interest and a second location within the cell. The ROIs are highlighted in (b) and (c). Each trace was offset for clarity.

In order to demonstrate a potential experimental scenario where a multimodal system would be beneficial to the user, hESC-cardiomyocytes were cultured on gold surfaces. First, bright-field microscopy was used to locate a hESC-CM; see figure 9(a). The periodic contractions of a living, beating cell could be visualized by monitoring the change in SPR intensity on the EPIX camera. By taking the difference of each subsequent frame with the first frame, a stack was produced that shows the area where there is the greatest variation in light intensity (‘hot-spot’); see figures 9(c) and (d). The background was corrected for by taking the difference of the ‘hot-spot’ with a sample of the rest of the cell which was not moving.

Figure 9(e) shows that the SPR intensity changes in time with the contractions of the stem cell-derived cardiomyocyte (‘signal’) with a high signal-to-noise ratio. Two control ROIs are plotted. One on the background and a secondary location within the cell. The resulting SPR responses show that only a couple of areas are moving within the cell, with the rest being uniform. The area where there is the greatest change in light intensity appears to be around the nucleus of the cell (see supplementary video 1). It is possible that the source of the signal is from the stress fibres or contractile apparatus within the hESC-CM pressing on the nucleus causing it to move. The observed contraction localized in the centre of the cell is typical for hESC-CM’s attached to rigid surfaces [48]. The control ROI on the ‘background’ was taken directly next to the beating cardiomyocyte, which shows no change in relative SPR intensity. This demonstrates that the change in SPR intensity is localized to the cell of interest and therefore, most likely due to refractive index changes or localized movement within the cell and not from movement causing mechanical waves throughout the volume. To fully utilize the multi-modal system, fluorescent labelling could be used for comparison of the label-free results with well-established labelling techniques.

## 5. Conclusions

In this paper, we have presented a multimodal platform for cell physiology combining SPR imaging with a number of ancillary microscopy systems. We have shown that the system is capable of both structural and functional imaging of cultured cells. Using the system for structural imaging, a number of modalities can be exploited including reflection/transmission microscopy, TIR micrscopy, epi-fluorescence and SPR imaging to obtain complementary information. A wide field-of-view has been demonstrated with a suitable spatial resolution for imaging cardiomyocytes and resolving individual axons and dendrites in cultured primary neurons label-free.

The ability of the system to study spatiotemporal cellular functions was demonstrated by imaging localized contractions of stem cell-derived cardiomyocytes. To the best of the author’s knowledge, this is the first demonstration of functional imaging of the refractive index changes using SPR in single cardiomyocytes. Using SPR could allow the localized contractions of cardiomyocytes to be imaged in real-time and drugs to be tested *in vitro* providing additional information compared to traditional non-imaging techniques [49, 50] and less computation compared to video microscopy [51].

In future work, a small network of cultured neurons will be grown on the surface of a sensor and any small changes to this light that occur during an action potential will be monitored.

## References

[1] Cohen L B, Keynes R D, Hille B (1968). Light scattering and birefringence changes during nerve activity. Nature.

[2] Stepnoski R A, LaPorta A, Raccuia-Behling F, Blonder G E, Slusher R E, Kleinfeld D (1991). Noninvasive detection of changes in membrane potential in cultured neurons by light scattering. Proc. Natl Acad. Sci..

[3] Shaked N T, Satterwhite L L, Bursac N, Wax A (2010). Whole-cell-analysis of live cardiomyocytes using wide-field interferometric phase microscopy. Biomed. Opt. Express.

[4] Le Ru E, Etchegoin P (2008). Principles of Surface-Enhanced Raman Spectroscopy: And Related Plasmonic Effects.

[5] Homola J, Yee S S, Gauglitz G (1999). Surface plasmon resonance sensors. Sensors Actuators B.

[6] Wakamatsu T, Aizawa K (2005). Penetration-depth characteristics of evanescent fields at metal attenuated total reflection. Japan. J. Appl. Phys..

[7] Peterson A W, Halter M, Tona A, Bhadriraju K, Plant A L (2009). Surface plasmon resonance imaging of cells and surface-associated fibronectin. BMC Cell Biol..

[8] Peterson A W, Halter M, Tona A, Plant A L (2014). High resolution surface plasmon resonance imaging for single cells. BMC Cell Biol..

[9] Yanase Y, Hiragun T, Ishii K, Kawaguchi T, Yanase T, Kawai M, Sakamoto K, Hide M (2014). Surface plasmon resonance for cell-based clinical diagnosis. Sensors.

[10] Toma K, Kano H, Offenhäusser A (2014). Label-free measurement of cell-electrode cleft gap distance with high spatial resolution surface plasmon microscopy. ACS Nano.

[11] Kim S A, Byun K M, Lee J, Kim J H, Kim D-G A, Baac H, Shuler M L, Kim S J (2008). Optical measurement of neural activity using surface plasmon resonance. Opt. Lett..

[12] Homola J (2008). Surface plasmon resonance sensors for detection of chemical and biological species. Chem. Rev..

[13] Abadian P N, Kelley C P, Goluch E D (2014). Cellular analysis and detection using surface plasmon resonance techniques. Anal. Chem..

[14] Nguyen H H, Park J, Kang S, Kim M (2015). Surface plasmon resonance: a versatile technique for biosensor applications. Sensors.

[15] Maier S A (2007). Plasmonics: Fundamentals and Applications.

[16] Otto A (1968). Excitation of nonradiative surface plasma waves in silver by the method of frustrated total reflection. Z. Phys..

[17] Kretschmann E, Raether H (1968). Radiative decay of non radiative surface plasmons excited by light. Z. Nat.forsch. A.

[18] Huang B, Yu F, Zare R N (2007). Surface plasmon resonance imaging using a high numerical aperture microscope objective. Anal. Chem..

[19] Stabler G, Somekh M G, See C W (2004). High-resolution wide-field surface plasmon microscopy. J. Microsc..

[20] Owega S, Poitras D (2007). Local similarity matching algorithm for determining SPR angle in surface plasmon resonance sensors. Sensors Actuators B.

[21] Sepúlveda B, Calle A, Lechuga L M, Armelles G (2006). Highly sensitive detection of biomolecules with the magneto-optic surface-plasmon-resonance sensor. Opt. Lett..

[22] Campbell C T, Kim G (2007). SPR microscopy and its applications to high-throughput analyses of biomolecular binding events and their kinetics. Biomaterials.

[23] Abayzeed S A, Smith R J, Webb K F, Somekh M G, See C W (2017). Sensitive detection of voltage transients using differential intensity surface plasmon resonance system. Opt. Express.

[24] Abayzeed S A, Smith R J, Webb K F, Somekh M G, See C W (2016). Responsivity of the differential-intensity surface plasmon resonance instrument. Sensors Actuators B.

[25] Wu S Y, Ho H P, Law W C, Lin C, Kong S K (2004). Highly sensitive differential phase-sensitive surface plasmon resonance biosensor based on the Mach–Zehnder configuration. Opt. Lett..

[26] Tan H M, Pechprasarn S, Zhang J, Pitter M C, Somekh M G (2016). High resolution quantitative angle-scanning widefield surface plasmon microscopy. Sci. Rep..

[27] Robertson S K, Bike S G (1998). Quantifying cell-surface interactions using model cells and total internal reflection microscopy. Langmuir.

[28] Funatsu T, Harada Y, Tokunaga M, Saito K, Yanagida T (1995). Imaging of single fluorescent molecules and individual ATP turnovers by single myosin molecules in aqueous solution. Nature.

[29] Axelrod D (2001). Total internal reflection fluorescence microscopy in cell biology. Traffic.

[30] Fish K N (2009). Total internal reflection fluorescence (TIRF) microscopy. Currr. Protoc. Cytom..

[31] Bauch M, Toma K, Toma M, Zhang Q, Dostalek J (2014). Plasmon-enhanced fluorescence biosensors: a review. Plasmonics.

[32] Keller H E (2003). Proper alignment of the microscope. Methods in Cell Biology.

[33] Hitzenberger C K, Danner M, Drexler W, Fercher A F (1999). Measurement of the spatial coherence of superluminescent diodes. J. Mod. Opt..

[34] Deng Y, Chu D (2017). Coherence properties of different light sources and their effect on the image sharpness and speckle of holographic displays. Sci. Rep..

[35] Dainty J C (2013). Laser Speckle and Related Phenomena.

[36] Foust A J, Schei J L, Rojas M J, Rector D M (2008). *In vitro* and *in vivo* noise analysis for optical neural recording. J. Biomed. Opt..

[37] Goss C A, Charych D H, Majda M (1991). Application of (3-mercaptopropyl) trimethoxysilane as a molecular adhesive in the fabrication of vapor-deposited gold electrodes on glass substrates. Anal. Chem..

[38] Aouani H, Wenger J, Gérard D, Rigneault H, Devaux E, Ebbesen T W, Mahdavi F, Xu T, Blair S (2009). Crucial role of the adhesion layer on the plasmonic fluorescence enhancement. ACS Nano.

[39] Palyvoda O, Chen C, Auner G (2007). Culturing neuron cells on electrode with self-assembly monolayer. Biosens. Bioelectron..

[40] Burridge P W, Anderson D, Priddle H, Barbadillo Muñoz M D, Chamberlain S, Allegrucci C, Young L E, Denning C (2007). Improved human embryonic stem cell embryoid body homogeneity and cardiomyocyte differentiation from a novel v-96 plate aggregation system highlights interline variability. Stem Cells.

[41] Kaech S, Banker G (2006). Culturing hippocampal neurons. Nat. Protoc..

[42] Born M, Wolf E (2013). Principles of Optics: Electromagnetic Theory of Propagation, Interference and Diffraction of Light.

[43] Meitzen J, Pflepsen K R, Stern C M, Meisel R L, Mermelstein P G (2011). Measurements of neuron soma size and density in rat dorsal striatum, nucleus accumbens core and nucleus accumbens shell: differences between striatal region and brain hemisphere, but not sex. Neurosci. Lett..

[44] Göktepe S, Abilez O J, Parker K K, Kuhl E (2010). A multiscale model for eccentric and concentric cardiac growth through sarcomerogenesis. J. Theor. Biol..

[45] von Keyserlingk Graf D, Schramm U (1984). Diameter of axons and thickness of myelin sheaths of the pyramidal tract fibres in the adult human medullary pyramid. Anat. Anz..

[46] Izzard C S, Lochner L R (1976). Cell-to-substrate contacts in living fibroblasts: an interference reflexion study with an evaluation of the technique. J. Cell Sci..

[47] Meyer R A (1979). Light scattering from biological cells: dependence of backscatter radiation on membrane thickness and refractive index. Appl. Opt..

[48] Kijlstra J D, Hu D, van der Meer P, Domian I J (2017). Single-cell functional analysis of stem-cell derived cardiomyocytes on micropatterned flexible substrates. Curr. Protoc. Stem Cell Biol..

[49] Jonsson M K B, Wang Q-D, Becker B (2011). Impedance-based detection of beating rhythm and proarrhythmic effects of compounds on stem cell-derived cardiomyocytes. Assay Drug Dev. Technol..

[50] Ferrie A M, Wu Q, Deichmann O D, Fang Y (2014). High frequency resonant waveguide grating imager for assessing drug-induced cardiotoxicity. Appl. Phys. Lett..

[51] Ahola A, Kiviaho A L, Larsson K, Honkanen M, Aalto-Setälä K, Hyttinen J (2014). Video image-based analysis of single human induced pluripotent stem cell derived cardiomyocyte beating dynamics using digital image correlation. Biomed. Eng. Online.

